# The association between multiple long‐term conditions and dementia: A UK cohort study

**DOI:** 10.1002/dad2.70230

**Published:** 2025-12-15

**Authors:** Hilary Shepherd, Adam Todd, David R. Sinclair, Charlotte L. Richardson, Fiona E. Matthews, Andrew Kingston

**Affiliations:** ^1^ Population Health Science Institute Newcastle University Newcastle upon Tyne UK; ^2^ Clinical Practice Research Datalink London UK; ^3^ School of Pharmacy Newcastle University Newcastle upon Tyne UK; ^4^ National Institute for Health and Care Research—Newcastle Patient Safety Research Collaborative Newcastle University Newcastle upon Tyne UK; ^5^ Institute for Clinical and Applied Health Research University of Hull Hull UK

**Keywords:** competing risk of death, Cox regression, electronic health‐care records, multimorbidity, multiple long‐term conditions

## Abstract

**INTRODUCTION:**

Studies examining the risk of dementia in people with multimorbidity are commonly conducted in research cohorts or outside the UK. Multimorbidity has historically been associated with aging, but recent research suggests that more than half of incidence cases occur in adults < 50.

**METHODS:**

Using UK primary care data, adjusted Cox regressions and competing risk of death models were used to determine risk of dementia in people with multimorbidity overall and by body system.

**RESULTS:**

People with multimorbidity had a greater risk of dementia that those without multimorbidity (hazard ratio [HR] = 4.01, 95% confidence interval [CI] 3.94–4.07). Among people with multimorbidity, the risk was highest for those when a neurological condition was included (HR = 2.19, 95% CI 2.15–2.23).

**DISCUSSION:**

Managing multimorbidity, particularly neurological conditions, is key and could delay or reduce the risk of dementia.

**Highlights:**

- People with multimorbidity experienced a greater risk of dementia than those without.
- Neurological multimorbidity presented the highest risk of dementia.
- Risk of dementia increased progressively with younger‐onset multimorbidity.
- Preventing or managing multimorbidity effectively could reduce or delay dementia.

## 1 BACKGROUND

Multimorbidity is usually defined as two or more long‐term conditions occurring within the same patient at the same time,^1^ and the global prevalence has been estimated to be 37.2%.^2^ To date, multimorbidity has been considered a condition of aging;^3^, ^4^ however, recent research has suggested that multimorbidity is increasingly occurring in younger people. Head et al.^5^ reported that more than half of the incidence of multimorbidity in England each year has occurred in adults < 50 years old. If this trend continues, multimorbidity onset may frequently precede a dementia diagnosis, making it an important factor in future dementia risk‐prevention strategies.

Dementia is an umbrella term describing various brain diseases which can affect memory, behavior, and the ability to complete everyday tasks.^6^ It has been estimated that, in 2019, 57.4 million individuals were living with dementia globally,^7^ including 885,000 people in the UK. By 2050, this is expected to increase to 152.8 million across the world.^8^ Research has highlighted that some health conditions including depression, hearing loss, and hypertension can individually contribute to increased risk of dementia^9^; there have been fewer studies examining how combinations of health conditions impact dementia risk due to the complexity of compiling a comprehensive list of conditions and how to define each one.

Globally, studies which have investigated this relationship have found overall multimorbidity is associated with an increased risk of dementia of up to 3.5 times but reported mixed results when investigating which types of conditions may be driving this, such as multimorbidity related to pain, cancer, or in the cardiometabolic or respiratory body systems.^10^, ^11^, ^12^, ^13^, ^14^, ^15^, ^16^, ^17^, ^18^, ^19^ Additionally, most of these studies use research cohorts except for two^11^, ^18^ which use national registers; include number of conditions varying from 3 to 60; and number of participants ranging from 2622^11^, ^17^ to 2,665,874.^18^ Of those conducted in the UK, three used research cohorts^10^, ^17^, ^20^ which often comprise participants who are healthier than average^21^ and frequently rely on self‐ or proxy‐reported outcomes. There has been limited research conducted using a representative real‐world UK‐based data source in which conditions are clinician diagnosed.

The combination of rising multimorbidity in younger people and existing associations between individual health conditions and dementia may result in a greater risk of dementia in a greater number of younger people, and this warrants further investigation. This research aims to (1) determine the risk of dementia in people with multimorbidity, and (2) assess how risk varies according to the body system affected.

## 2 METHODS

### 2.1 Data source

The data for this study are from the March 2023 snapshot of Clinical Practice Research Datalink (CPRD) GOLD, a pseudonymized database of primary care records from general practitioners (GP) in the UK, which is representative in terms of age, sex, and ethnicity.^22^ The snapshot contained 21,176,443 patients from 986 GP practices^23^ and all their medical events. These primary care data were linked to both patient‐level and practice‐level Index of Multiple Deprivation (IMD) to provide an area‐level deprivation score for every person based on the characteristics of small neighborhoods. The IMD score can be generated at patient level (where the patient lives) or at the practice level (the location of the GP practice), and is calculated based on income, employment, education and skills, health, housing, crime, access to services, and living environment. Each person is assigned a relative deprivation score, and for this study, IMD was calculated in deciles from 1 (least deprived) to 10 (most deprived). Medical history for each patient was followed until the patient de‐registered from the GP practice, or the practice stopped contributing data to CPRD, whichever was earlier. The scientific protocol was approved by CPRD (protocol number 23_003176) and accessed via the annual license of Newcastle University. The study was conducted according to Strengthening the Reporting of Observational Studies in Epidemiology (STROBE) guidelines (Supplementary Material A in supporting information).

**RESEARCH IN CONTEXT**

1. **Systematic review**: A literature review highlighted that the onset of multimorbidity (two or more long‐term conditions) is rising in younger people. The association between multimorbidity and dementia had not been thoroughly investigated in the UK using routinely collected data, using data which prioritizes clinician diagnosed conditions over self‐reported ones.
2. **Interpretation**: People with multimorbidity were at greater risk of dementia than people without multimorbidity. People with neurological conditions were at greatest risk, and more conditions at younger ages resulted in greater risk. Managing long‐term conditions could delay or reduce the risk of dementia.
3. **Future directions**: Future research could investigate common disease trajectories which lead to dementia and the differences in risk for population subgroups.

### 2.2 Data cleaning and source population

To define usable patient records, patients with missing or undefined sex (*n* = 1113), with no follow‐up time (*n* = 149), and those < 40 years old by follow‐up end (*n* = 11,267,869) were excluded, as patients were unlikely to have developed either multimorbidity^1^ or dementia^24^ before this age. Patients registered at GP practices outside of England (*n* = 3,759,043) were excluded as IMD data are only available for England. Patients contributing < 1 day of follow‐up time (*n* = 63,062) and those who registered at their GP practice before 1912 (*n* = 6638) were also excluded as they preceded the creation of the National Insurance Act (the predecessor of the National Health Service [NHS]) and the allocation of patients to individual GPs, and therefore may be unreliable. This resulted in 6,078,569 patients for inclusion, each with individual periods of medical history of varying durations. These criteria ensured no missing data for sex, year of birth, IMD, follow‐up start, or follow‐up end.

**FIGURE 1:**
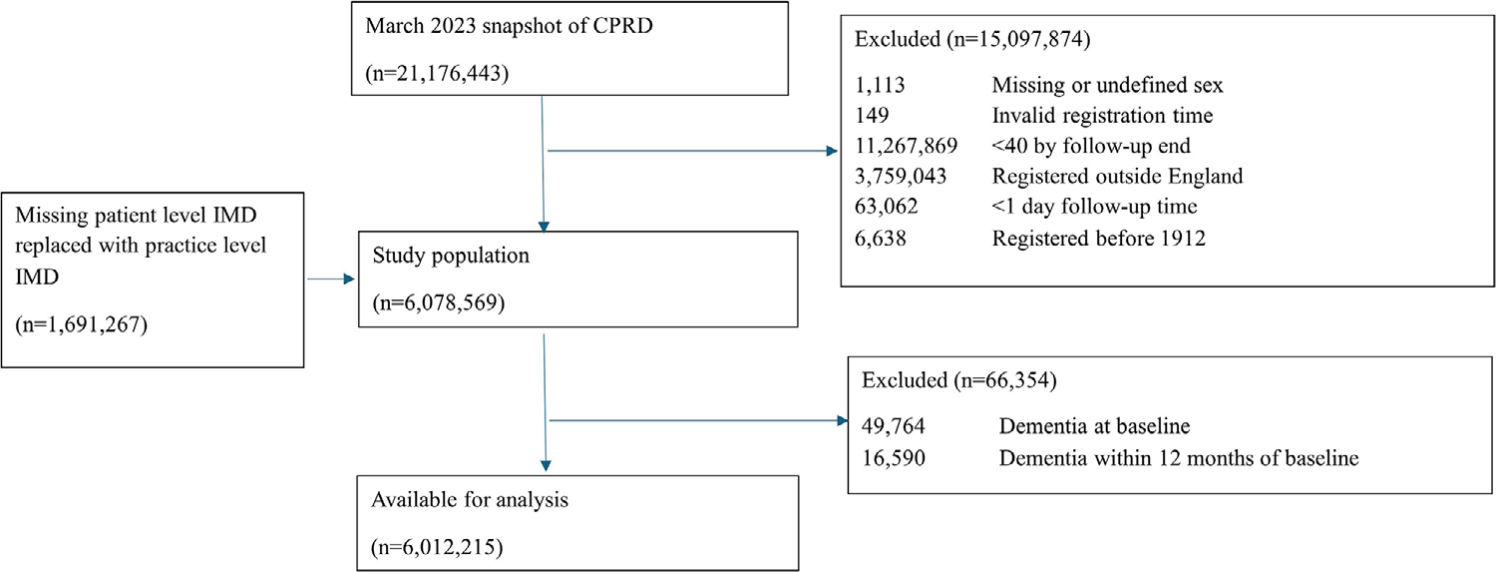
Flow diagram of study population (*n* refers to distinct patients). CPRD, Clinical Practice Research Datalink; IMD, Index of Multiple Deprivation.

Prior to analysis, people with existing dementia at baseline (*n* = 49,973), or who developed dementia in the 12 months after baseline (*n* = 16,618) were excluded to mitigate risk of reverse causality between multimorbidity conditions and dementia (see Figure 1).

All diagnoses occurring before follow‐up end were considered. Any diagnoses with missing associated dates were dropped, resulting in no missing data for the date of exposure or outcome.

When patient‐level IMD was missing, it was replaced with practice‐level IMD (*n* = 1,691,267 records; 27.8%). Deciles of IMD were aggregated into three levels: least deprived (IMD levels 1–2), middle 60% (IMD levels 3–8), and most deprived (IMD levels 9–10) to elucidate effects associated with the highest and lowest boundaries of deprivation.

### 2.3 Definition of exposure

Multimorbidity was defined as a count of two or more of 36 chronic conditions (not including dementia) based on methodology by Barnett et al.,^1^ Cassell et al.,^3^ and Kuan et al.^25^ The list of conditions proposed by Barnett is used widely in multimorbidity research^3^, ^26^, ^27^, ^28^ including in the generation of the Cambridge Multimorbidity Score, which was subsequently validated in the UK Biobank.^29^ The code lists used for each condition in this study were created and validated by researchers at Cambridge Primary Care Unit (PCU),^30^ including four GPs, and were updated when necessary prior to use in this study (see Supplementary Material B in supporting information).

All code lists are available at https://github.com/hilarylshepherd/CPRD_multimorbidity_codelists.

### 2.4 Definition of outcome

The GP‐validated code list by Cambridge PCU was also used to define all‐cause dementia in the study data and includes Alzheimer's disease and vascular dementia. It was also expanded to include terms relating to frontal lobe, frontotemporal, organic psychotic, Lewy body, Pick's disease, presenile, and Parkinson's disease dementia (see Supplementary Material C in supporting information).

### 2.5 Covariates

Covariates adjusted for in the analyses were age, sex (male or female), deprivation (using three levels of IMD), and residual multimorbidity. Residual multimorbidity was defined as the count of multimorbidity conditions per person at the specified time point, excluding the one of interest, to isolate the association of an individual condition as much as possible. Age was calculated using year of birth only, as month is not available for every patient in the CPRD data, and day is not provided at all. Ethnicity was explored as a covariate for inclusion but discarded due to 70% missing data. Similarly, lifestyle covariates such as smoking^31^ and body mass index (BMI)^32^ may not be reliably recorded between patients or over time. Alcohol consumption was not included to avoid collinearity, as one of the multimorbidity conditions of interest was alcohol misuse disorder.

### 2.6 Statistical analyses

Index date for each condition was the first ever valid occurrence of the relevant code in the medical history. Index date for multimorbidity was equal to the index date of the second individual condition—when this was on or before baseline, individuals were considered to have multimorbidity at baseline. Baseline for each patient was considered the date at which they registered at their GP practice. To determine risk of dementia by body system, all 36 conditions were organized into 1 of 12 categories based on the primary body system affected (see Supplementary Material D in supporting information).

All statistical analyses were conducted using Stata 18 and R version 4.4.0. Cox regressions were used to investigate risk of dementia, and the proportional hazards assumptions were tested based on Schoenfeld residuals. Modeling the cumulative incidence function using the Fine–Gray method was conducted to account for the competing risk of death. As CPRD captures only parts of each person's life (i.e., left‐censoring at GP registration and right‐censoring at GP de‐registration or last data collection from the practice), time at risk was measured from registration with the GP (baseline, *t_0_*) to the earliest dementia diagnosis or end of follow‐up (*t_1_*). To ensure that results were robust and to avoid bias from unequal follow‐up time, we ran a landmark analysis at fixed ages, including a sensitivity analysis on only those who were alive and under observation at each specified age.^33^ In this study, the landmark time points were ages: every 5 years from 55 to 90. Therefore, only patients who were alive and contributing data to their GP practice at the designated “landmark age” were analyzed. Landmark analyses help mitigate immortal time bias, which arises when, due to exposure definition or study design, there is a period over which the outcome cannot occur.^34^ In this context, this may happen as patients are required to survive until their GP registration date so they can enter the study, potentially biasing the sample toward healthier patients. By estimating the risk only in those alive and under observation at each landmark age, the analyses provide age‐specific estimates and reduce bias from unequal survival time. The risk of dementia in patients with multimorbidity at each of these landmark ages was calculated compared to patients without multimorbidity.

## 3 RESULTS

The study population included 6,078,569 people. Those with existing dementia at (*n* = 49,764) or within 12 months of (*n* = 16,590) baseline were excluded, leaving 6,012,215 patient records available for analysis (Table 1). These were split evenly by sex (51% female) and on average were 43.8 years old at baseline (standard deviation [SD] 19.3), but people with multimorbidity at baseline were on average older than those without (56.9 [SD 18.9] vs. 41.7 [SD 18.5]).

**TABLE 1:** Description of study population at baseline by multimorbidity status at baseline.

Demographic	No multimorbidity	Multimorbidity
N	5,165,125 (85.9%)	847,090 (14.1%)
Sex		
Male	2,583,147 (50.0%)	362,471 (42.8%)
Female	2,581,978 (50.0%)	484,619 (57.2%)
Age at baseline in years (mean, SD)	41.7 (18.5)	56.9 (18.9)
Years of follow‐up time (mean, SD)	16.4 (15.0)	12.5 (12.8)
Region of England		
North East	87,269 (1.7%)	14,427 (1.7%)
North West	704,179 (13.6%)	123,502 (14.6%)
Yorkshire & The Humber	234,789 (4.5%)	28,900 (3.4%)
East Midlands	237,929 (4.6%)	24,912 (2.9%)
West Midlands	561,191 (10.9%)	99,910 (11.8%)
East of England	562,574 (10.9%)	81,693 (9.6%)
London	880,795 (17.1%)	135,078 (15.9%)
South East	1,355,867 (26.3%)	250,640 (29.6%)
South West	540,532 (10.5%)	88,028 (10.4%)
Patient‐level deprivation		
Deciles 1 + 2: least deprivation	1,083,423 (21.0%)	181,080 (21.4%)
Deciles 3–8: middle 60%	3,159,570 (61.2%)	510,874 (60.3%)
Deciles 9 + 10: most deprivation	922,132 (17.9%)	155,136 (18.3%)

Abbreviation: SD, standard deviation.

### 3.1 Risk of dementia in people with multimorbidity

In the Cox models (adjusted for sex, year of birth, and deprivation), there was a positive association between multimorbidity and risk of dementia (hazard ratio [HR] = 1.93, 95% confidence interval [CI] 1.89–1.96) compared to people without multimorbidity (Table 2). The association remained after adjusting for the competing risk of death (HR = 4.01, 95% CI 3.94–4.07) and when considering only multimorbidity which was already present at baseline (adjusted HR = 2.51, 95% CI 2.44–2.59).

**TABLE 2:** Risk of dementia in people with multimorbidity.

Exposure	Cox regression Unadjusted HR [95% CI]	Cox regression Adjusted HR [95% CI]	Competing risk of death Adjusted HR [95% CI]
Multimorbidity (2+ conditions)	2.36 [2.31, 2.39]	1.93 [1.89, 1.96]	4.01 [3.94, 4.07]

*Note*: Exponentiated coefficients; 95% confidence intervals in brackets. Adjusted model includes sex, year of birth, and deprivation. Compared to people without multimorbidity (0–1 conditions).

Abbreviations: CI, confidence interval; HR, hazard ratio.

### 3.2 Body system with greatest risk of dementia

**FIGURE 2:**
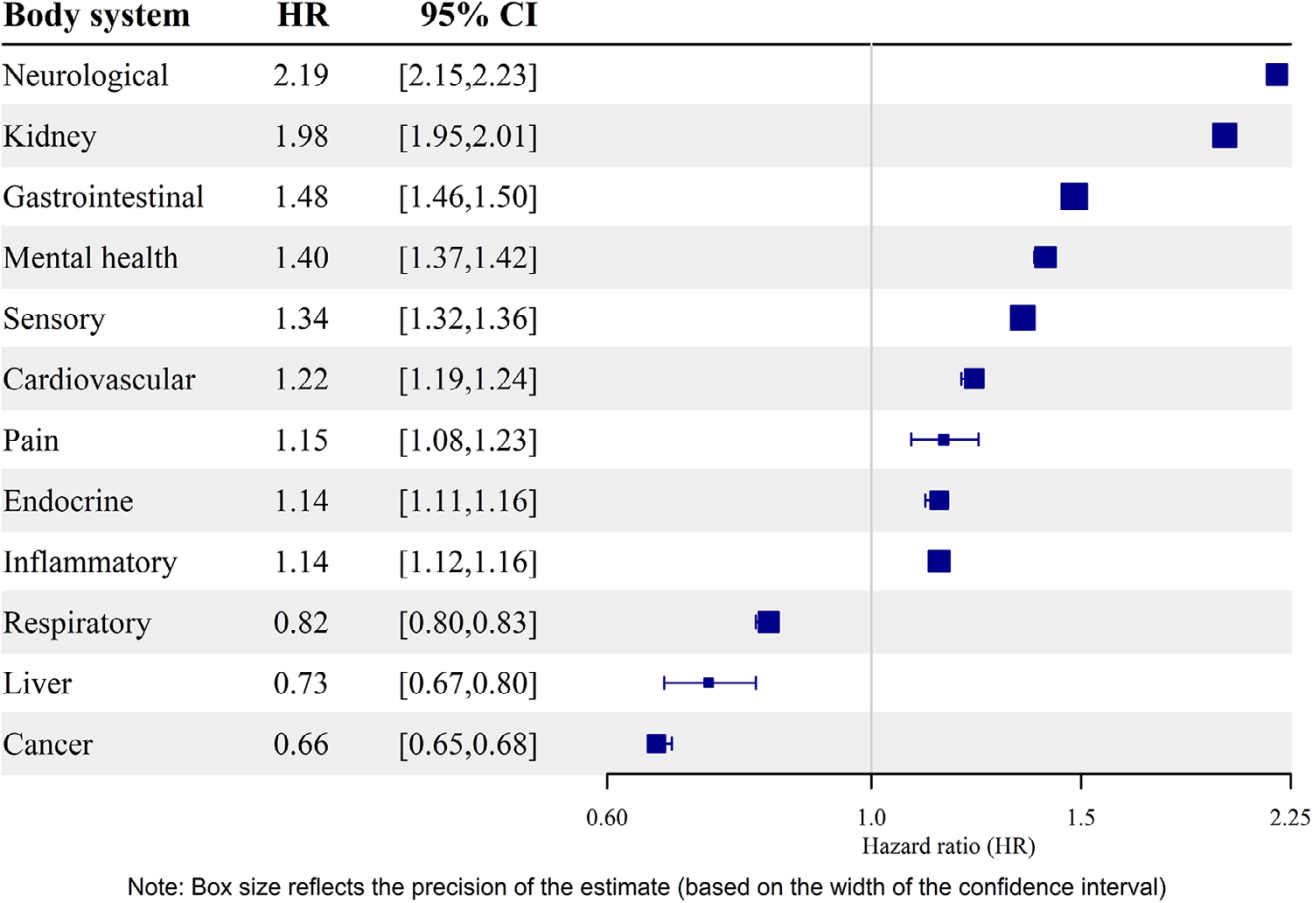
Risk of dementia by body system, competing risk of death model adjusted for sex, year of birth, deprivation, and residual multimorbidity. CI, confidence interval; HR, hazard ratio.

When grouped by body system, multimorbidity which included at least one condition in the neurological body system presented more than twice the risk of dementia compared to multimorbidity that did not (HR = 2.19, 95% CI 2.15–2.23; Figure 2).

Cancer, as a body system, presented a consistently low risk in the Cox model (HR = 0.77, 95% CI 0.76–0.79) and in the competing risk of death model (HR = 0.66, 95% CI 0.64–0.67) compared to other body systems (Supplementary Material E in supporting information), and as a single condition compared to other single conditions (Supplementary Material F in supporting information). The risk of dementia in multimorbidity that included sensory and kidney body systems were most impacted by the competing risk of death (Supplementary Material E). Descriptions of the populations who had multimorbidity in each body system are presented in Supplementary Material G in supporting information.

### 3.3 Sensitivity analysis

We conducted a sensitivity analysis to investigate whether the overall number of conditions in people with multimorbidity changed the risk of dementia. After accounting for the competing risk of death, there was evidence of a dose–response relationship, whereby the number of conditions increased risk. It also indicated that individuals accumulating > 4 conditions were at higher risk of death before developing dementia, and the same pattern, but of increased magnitude, was seen when considering only conditions already present at baseline (Supplementary Material H in supporting information).

Landmark analysis, using age as the landmark, was also conducted to determine dementia risk in multimorbidity at various ages, to mitigate the risk of immortal time bias by requiring every person to have contributed the same amount of time to the analysis. Risk of dementia was calculated at 5‐year intervals from age 55 to 90, resulting in evidence of a dose–response relationship, with greater risk at younger ages. The association was strengthened after adjustment for competing risk of death, indicating that people with multimorbidity at each landmark age have a greater risk of dementia compared to those without multimorbidity at that age, but that risk of death in this group is prevalent and masking the association in the Cox model (Table 3).

**TABLE 3:** Landmark analysis for risk of dementia at each age.

Landmark age	Cox regression Adjusted HR [95% CI]	Competing risk of death Adjusted HR [95% CI]
55	3.11 [2.73, 3.54]	3.80 [3.35, 4.31]
60	2.58 [2.36, 2.82]	3.54 [3.25, 3.85]
65	2.15 [2.02, 2.29]	3.28 [3.09, 3.48]
70	1.91 [1.83, 2.00]	3.07 [2.97, 3.18]
75	1.73 [1.68, 1.79]	2.80 [2.72, 2.89]
80	1.56 [1.53, 1.60]	2.65 [2.60, 2.71]
85	1.45 [1.43, 1.48]	2.51 [2.47, 2.55]
90	1.36 [1.34, 1.39]	2.47 [2.44, 2.50]

*Note*: Exponentiated coefficients; 95% confidence intervals in brackets. Both models adjusted model adjusted for sex, year of birth, deprivation, and residual multimorbidity. Compared to no multimorbidity at landmark age.

Abbreviations: CI, confidence interval; HR, hazard ratio.

## 4 DISCUSSION

For the first objective of this study, we found an increased risk of dementia in people with multimorbidity compared to those without (HR = 4.01, 95% CI 3.94–4.07). For the second objective, we found that risk was highest for people with multimorbidity that included the neurological body system (HR = 2.19, 95% CI 2.15–2.23). Sensitivity analysis confirmed that risk of dementia increased as overall burden of multimorbidity increased (2 conditions HR = 2.64, 95% CI 2.58–2.70 vs. 5+ conditions HR = 5.27, 95% CI 5.17–5.36), and that risk was highest for younger people (multimorbidity at age 55 HR = 3.62, 95% CI 3.21–4.08 vs. multimorbidity at age 90 HR = 2.47, 95% CI 2.44–2.50).

The risk of dementia in this study is higher than previous evidence which estimates the risk to lie between 1.15^13^ and 2.59.^19^ The substantial increase observed after competing risk of death analysis (from 1.93 to 4.01) indicates that death has a substantial impact on the likelihood of observing dementia in this study population. Of the previous studies which have investigated this association, only two included competing risk of death analyses^18^, ^19^ and neither were conducted on a UK population. Use of a real‐world data sources may also account for the higher estimate found in this study, as it may be that research cohorts used in other studies comprise a healthier population on average than the general population. Clinician‐coded multimorbidity may result in stronger observed associations compared to self‐reported data due to diagnostic accuracy. Alternatively, patients with multimorbidity in our study may have been diagnosed with dementia earlier due to an increased chance of clinical contact.

The link we observed between neurological conditions and dementia is consistent with existing literature,^35^ and the individual condition models (Supplementary Material F) indicated that learning disability (relating primarily to Down syndrome) presented with the greatest individual risk (HR = 7.50, 95% CI 6.89–8.16), another well‐established association.^36^ The risk associated with cancer we found was low and not explained by the competing risk of death (HR = 0.66, 95% CI 0.64–0.67), which is in line with evidence by Chen et al.^37^ and Ben Hassen et al.^10^ but contrary to Khondoker et al.^15^ Biological mechanisms for this “protective effect” have been discussed elsewhere, including the possibility that risk differs by dementia subtype.^38^, ^39^, ^40^, ^41^ It could indicate reverse causality for conditions in which prodromal states are long; however, this may equally be a survivor effect in which individuals who survive cancer treatment are predisposed to longer life,^42^ or an artefact of our multimorbidity definition which required two events to occur before dementia, as any events after dementia were excluded. This could also explain the low associations with the respiratory and cardiovascular body systems, as conditions in both categories are strongly associated with age.^43^, ^44^ The change in estimate for the kidney body system (from HR = 0.47, 95% CI 0.46–0.48 in the adjusted Cox model to HR = 1.98, 95% CI 1.94–2.01 following competing risk of death), indicates that patients with chronic kidney disease are at high risk of dying before onset of dementia. This pattern was reflected in the sensory body system, which has been found by other groups^9^; however, the current study cannot distinguish whether these sensory conditions are independent conditions or are symptoms of others, for example, diabetic retinopathy after diabetes. Body system groups were not mutually exclusive; therefore, one person may have conditions from several body systems simultaneously. In line with the landmark analysis, Ben Hassen et al.^10^ found that the risk of dementia increased with earlier onset of multimorbidity, though reported effects were weaker than those in the present study. However, all findings presented a consistent direction of effect, indicating that multimorbidity beginning earlier in life increases the risk of developing dementia later.

These findings highlight the need for effective prevention strategies for multimorbidity in mid‐life, and good management of existing multimorbidity from at least age 55. By preventing the development or worsening of multimorbidity, it may be possible to delay or reduce onset of dementia. Effective prevention would enable individuals to live longer in good health as well as reduce the family, social, and medical costs of dementia care.^45^ There is potential for individual and societal‐level impact if the development of a long‐term condition—linked to increased dementia risk—is delayed or avoided, for example, depression.^46^ Further research could estimate the impact of medication effects on individuals with multimorbidity (and harmful effects if it results in polypharmacy), the ordering of disease accumulation,^47^ common trajectories of disease, and risk differences by population subgroups.

### 4.1 Strengths and limitations

This study has many strengths. The CPRD data source is a large database of primary care data, broadly representative of the UK in terms of age, ethnicity, and sex.^22^ The size and availability of long‐term follow‐up in CPRD allows for robust observational studies with confidence that results are generalizable to England. As dementia is generally diagnosed and managed in primary care, CPRD is highly suitable for this investigation. While any diagnoses of dementia made outside primary care (and not reported to the GP) will be missing, recent work has determined that dementia recorded in NHS hospitals is in accordance with primary care records.^48^

There are several limitations to note. The absence of lifestyle covariates such as smoking, BMI, diet, and physical activity may have led to residual confounding, likely biasing estimates upward because these factors are associated with both multimorbidity and dementia risk.

This study was not representative geographically, as some regions were under‐represented (Yorkshire & Humber, East of England) or over‐represented (London, South East), and patients living in the most deprived areas were under‐represented. As GPs are invited to participate in data linkage on behalf of their practice, there may be bias among the GPs who contribute. Similarly, this study may generate bias by under‐representing people with dementia in the community who do not visit the doctor. We did not examine covariates such as smoking status or BMI as the data may not be reliably recorded,^31^, ^32^ which could have biased the sample toward a healthier population or introduce unmeasured confounding.

Also, although we used an established definition of multimorbidity and validated code lists, it may miss important conditions. Presence of conditions was based on codes, and missing codes assumes the condition is not present. Further, changing thresholds of some conditions within the Quality Outcomes Framework (QOF) may have affected the recording practices of GPs. The QOF is an incentive program which rewards GPs for good practice, and GPs score points for meeting criteria. However, criteria can change over time—for example, the threshold for a diagnosis of hypertension changed from 150/90 mmHg in 2009/2010^49^ to 140/90 mmHg in 2022/2023,^50^ which is not accounted for in this study. In the Cox regression models, including the year of birth violated the proportional hazards assumption that risk remained constant over time. To account for this, age was used as the underlying time scale, as a more appropriate way to account for the time‐related risk of dementia. However, this too violated the proportional hazards assumption (though to a lesser extent), likely due to limited variance in the hazard function later in follow‐up. Visual inspection of the smoothed hazard function for the main analyses suggested that fewer dementia diagnoses were recorded later in follow‐up and could explain the reduced variance. As a result, the hazard ratios should be interpreted as average effects over the follow‐up period and not strictly as constant effects. The use of the landmark analyses, by age, also reduced the immortal time bias risk by providing clearer age‐specific risk estimates. Residual multimorbidity was not used in models containing number of conditions due to collinearity.

## 5 CONCLUSION

There is an increased risk of dementia in people with multimorbidity, particularly in people with neurological conditions and those who have higher overall numbers of conditions at younger ages. Prevention or better management of long‐term conditions has the potential to delay the onset of dementia or reduce the number of people developing dementia. Future research could focus on the impact of medication, common trajectories of disease, and differences in risk for population subgroups.

## CONFLICT OF INTEREST STATEMENT

Hilary Shepherd works part time for Clinical Practice Research Datalink (CPRD). CPRD had no part in designing or conducting the study. All other authors report no conflicts of interest. Author disclosures are available in the supporting information.

## CONSENT STATEMENT

Individual consent was not necessary for this study because it is not possible to ascertain the identity of individual patients in any dataset that CPRD holds. CPRD never receives directly identifiable information such as name, address, NHS number, or date of birth from any data source.

## Supporting information



Supporting information

Supporting information
