# Supplementary material for: The association between multiple long‐term conditions and dementia: A UK cohort study
Source: Alzheimers Dement (Amst). 2025 Dec 15;17(4):e70230. doi: 10.1002/dad2.70230 (PMC12703650; doi:10.1002/dad2.70230)
Supplement: Supplementary file 1 — Supporting information [file DAD2-17-e70230-s001.docx]

# Supplementary material

## A

**Table A: STROBE checklist for cohort studies**

|  | Item No | Recommendation | Page No |
| --- | --- | --- | --- |
| **Title and abstract** | 1 | (*a*) Indicate the study’s design with a commonly used term in the title or the abstract |  |
|  |  | (*b*) Provide in the abstract an informative and balanced summary of what was done and what was found | 1 |
| Introduction | | | |
| Background/rationale | 2 | Explain the scientific background and rationale for the investigation being reported | 3 |
| Objectives | 3 | State specific objectives, including any prespecified hypotheses | 3 |
| Methods | | | |
| Study design | 4 | Present key elements of study design early in the paper | 4 |
| Setting | 5 | Describe the setting, locations, and relevant dates, including periods of recruitment, exposure, follow-up, and data collection | 4 |
| Participants | 6 | (*a*) Give the eligibility criteria, and the sources and methods of selection of participants. Describe methods of follow-up | 4 |
|  |  | (*b*) For matched studies, give matching criteria and number of exposed and unexposed |  |
| Variables | 7 | Clearly define all outcomes, exposures, predictors, potential confounders, and effect modifiers. Give diagnostic criteria, if applicable | 6 |
| Data sources/ measurement | 8 | For each variable of interest, give sources of data and details of methods of assessment (measurement). Describe comparability of assessment methods if there is more than one group | 6 |
| Bias | 9 | Describe any efforts to address potential sources of bias | 6 |
| Study size | 10 | Explain how the study size was arrived at | 4 |
| Quantitative variables | 11 | Explain how quantitative variables were handled in the analyses. If applicable, describe which groupings were chosen and why | 6 |
| Statistical methods | 12 | (*a*) Describe all statistical methods, including those used to control for confounding |  |
|  |  | (*b*) Describe any methods used to examine subgroups and interactions | 6 |
|  |  | (*c*) Explain how missing data were addressed |  |
|  |  | (*d*) If applicable, explain how loss to follow-up was addressed |  |
|  |  | (*e*) Describe any sensitivity analyses |  |
| Results | | |  |
| Participants | 13 | (a) Report numbers of individuals at each stage of study—e.g. numbers potentially eligible, examined for eligibility, confirmed eligible, included in the study, completing follow-up, and analysed | 5 |
|  |  | (b) Give reasons for non-participation at each stage |  |
|  |  | (c) Consider use of a flow diagram |  |
| Descriptive data | 14 | (a) Give characteristics of study participants (e.g. demographic, clinical, social) and information on exposures and potential confounders | 7 |
|  |  | (b) Indicate number of participants with missing data for each variable of interest |  |
|  |  | (c) Summarise follow-up time (e.g., average and total amount) |  |
| Outcome data | 15 | Report numbers of outcome events or summary measures over time |  |

| Main results | 16 | (*a*) Give unadjusted estimates and, if applicable, confounder-adjusted estimates and their precision (e.g., 95% confidence interval). Make clear which confounders were adjusted for and why they were included | 7-8 |
| --- | --- | --- | --- |
|  |  | (*b*) Report category boundaries when continuous variables were categorized |  |
|  |  | (*c*) If relevant, consider translating estimates of relative risk into absolute risk for a meaningful time period |  |
| Other analyses | 17 | Report other analyses done—e.g. analyses of subgroups and interactions, and sensitivity analyses | 9 |
| Discussion | | | |
| Key results | 18 | Summarise key results with reference to study objectives | 10 |
| Limitations | 19 | Discuss limitations of the study, taking into account sources of potential bias or imprecision. Discuss both direction and magnitude of any potential bias | 11-12 |
| Interpretation | 20 | Give a cautious overall interpretation of results considering objectives, limitations, multiplicity of analyses, results from similar studies, and other relevant evidence | 12 |
| Generalisability | 21 | Discuss the generalisability (external validity) of the study results | 11 |
| Other information | | | |
| Funding | 22 | Give the source of funding and the role of the funders for the present study and, if applicable, for the original study on which the present article is based | 12 |

## B

**Table B: Definitions of individual multimorbidity conditions**

| **Condition** | **Definition in this study** | **Cambridge PCU definition** |
| --- | --- | --- |
| Alcohol misuse | Medcode ever | n/a |
| Anorexia or bulimia | Medcode ever | n/a |
| Anxiety & other neurotic, stress related & somatoform disorders  Or  Depression* | Combined anxiety and depression medcode lists, included only patients with 3+ medcodes within any12 month period. First event considered index date. | Read code in last 12 months OR 4+ anxiolytic/hypnotic prescriptions in last 12 months (excluding SSRIs and tricyclics) |
| Asthma* | First medcode recorded >18 years old to capture historical adult-onset asthma | Read code ever recorded AND any prescription in last 12 months |
| Atrial fibrillation | Medcode ever | n/a |
| Blindness and low vision | Medcode ever | n/a |
| Bronchiectasis | Medcode ever | n/a |
| Cancer* | Medcode ever | New diagnosis in the last 5 years |
| Chronic kidney disease* | Diagnosis medcode ever OR eGFR reading of <60. Medcode list created. | The highest value of the last two eGFR readings is <60 mL/min. |
| Chronic Liver Disease and Viral Hepatitis | Medcode ever | n/a |
| Chronic sinusitis | Medcode ever | n/a |
| Constipation* | 3+ prodcodes within 12 months. | 4+ laxatives prescriptions in the last year |
| COPD | Medcode ever | n/a |
| Coronary heart disease | Medcode ever | n/a |
| Diabetes | Medcode ever | n/a |
| Diverticular disease of intestine | Medcode ever | n/a |
| Epilepsy * | Medcode ever | Read code ever recorded and antiepileptic prescription in the last 12 months |
| Hearing loss | Medcode ever | n/a |
| Heart failure* | Medcode ever. Code list expanded | n/a |
| Hypertension | Medcode ever | n/a |
| Inflammatory bowel disease | Medcode ever | n/a |
| Irritable bowel syndrome* | Medcode ever | Read code ever recorded or 4+ antispasmodic prescription only in the last 12 months |
| Learning disability | Medcode ever | n/a |
| Migraine* | 3+ medcodes within 12 months. Code list expanded | 4+ prescriptions only medicine anti-migraine prescriptions in the last year |
| Multiple sclerosis | Medcode ever | n/a |
| Painful condition* | Medcode for chronic pain ever. Code list expanded. | 4+ POM analgesics in the last 12 months OR 4+ anti-epileptics in last 12 months in the absence of an epilepsy read code |
| Parkinson's disease | Medcode ever | n/a |
| Peptic Ulcer Disease | Medcode ever | n/a |
| Peripheral vascular disease | Medcode ever | n/a |
| Prostate disorders | Medcode ever | n/a |
| Psoriasis or eczema* | 3+ medcodes within 12 months. Code list expanded | Read code ever recorded and 4+ related prescriptions in last 12 months (excluding simple emollients) |
| Psychoactive substance misuse | Medcode ever | n/a |
| Rheumatoid arthritis, other inflammatory polyarthropathies & systematic connective tissue disorders | Medcode ever | n/a |
| Schizophrenia (and related non-organic psychosis) or bipolar disorder* | Medcode ever | Read code ever recorded or lithium ever recorded |
| Stroke & transient ischaemic attack | Medcode ever | n/a |
| Thyroid disorders | Medcode ever | n/a |

Conditions marked (*) indicate where the definition from Cambridge Primary Care Unit was updated. “Medcode” represents the unique medical code used to represent events in CPRD. “Prodcode” represents product codes used to represent prescribed medicines in CPRD. Please note:

- We did not use the definition of “currently active” for any conditions (cancer, epilepsy, heart failure, irritable bowel syndrome) as this did not allow for specifically excluding events which occurred within the 12 months prior to dementia. Any events occurring in the 12 months prior to a dementia diagnosis were excluded in this study to mitigate the risk of reverse causality, i.e. events which may be linked to the prodromal stages of dementia. Excluding all “currently active” events, which could have occurred any time in the 5 years prior to dementia, was agreed as too conservative by the study team. Therefore, this expanded the time window to “ever” and included historical events, and “H/O” (history of) codes were retained in the code lists.
- Anxiety and depression were combined into one list because conditions were frequently diagnosed together e.g. Read code E200300 (medcode 655) “Anxiety with depression” associated with 1,162,844 clinical events and Read code Eu41211 (medcode 7749) “Mild anxiety depression” associated with 11,754 clinical events.
- For asthma, which is commonly resolved in childhood, we excluded events diagnosed before age of 18, according to the method of Head at al ^1^**.**
- Due to not using the definition of “currently active”, historical cancers were included
- To search for eGFR readings in CPRD GOLD, entity type 466 (“Glomerular Filtration Rate”) from the Test table was used. Any non-missing values <60 were considered a valid eGFR result indicating chronic kidney disease (equating to between moderate and very high risk according to NICE guidelines ^2^) and were appended to results form the medical code list indicating a diagnosis of chronic kidney disease. If two events were recorded on two different dates, the earliest was considered the index date.
- For constipation, 3 or more prescribed medications within any 12-month period was considered a chronic diagnosis, rather than just the last year, and the index date of the first prescription was considered index date for constipation to accurately reflect the length of time the condition was ongoing.
- For migraine, 3 or more medical diagnoses within any 12-month period was considered a chronic diagnosis, and product codes for anti-migraine medicines were not used to mitigate the risk that these were prescribed for another reason, as indication for medication is not available in the data. The index date of the first diagnosis was considered index date for migraine to accurately reflect the length of time the condition was ongoing.
- For defining a painful condition, a medical codelist relating to chronic pain was specifically created. As pain medications may relate to any condition, for acute or long-term reasons, and may also be used as an anti-epileptic medication, using product codes alone was agreed by the project team as not detailed enough information to diagnose “painful condition”. The difficulties with diagnosing this are also recognised in the documentation by Cambridge Primary Care Unit ^3^
- For psoriasis or eczema, 3 or more medical diagnoses within any 12-month period was considered a chronic diagnosis. The index date of the first diagnosis was considered index date for migraine to accurately reflect the length of time the condition was ongoing.
- For schizophrenia or bipolar disorder, any event ever recorded was considered a diagnosis. As lithium can indicate conditions outside of schizophrenia or bipolar disorder, including depressive episodes resistant to tricyclic antidepressants^4^, prescriptions of lithium were not included in the definition.
- Final code lists for all 37 conditions (36 individual multimorbidity conditions + dementia) can be viewed [here](https://github.com/hilarylshepherd/CPRD_multimorbidity_codelists).

References

1. Head A, Fleming K, Kypridemos C, Schofield P, Pearson-Stuttard J, O’Flaherty M (2021) Inequalities in incident and prevalent multimorbidity in England, 2004–19: a population-based, descriptive study. Lancet Healthy Longev 2:e489–e497

2. National Institute for Health and Care Excellence (2021) Classification of CKD in adults.

3. Cambridge Primary Care Unit (2022) Cambridge Primary Care Unit: Code Lists. https://www.phpc.cam.ac.uk/pcu/research/research-groups/crmh/cprd_cam/codelists/. Accessed 11 Jul 2023

4. Ercis M, Ozerdem A, Singh B (2023) When and How to Use Lithium Augmentation for Treating Major Depressive Disorder. Journal of Clinical Psychiatry. https://doi.org/10.4088/JCP.23AC14813

## C

**Table C: Definition of dementia**

| **Condition** | **Definition** | **Original definition** |
| --- | --- | --- |
| Dementia | Medcode ever recorded. Code list expanded. | n/a |

The code list for dementia was expanded from 53 to 75 codes based on terms and based on the hierarchical Read structure by locating codes with relevant character stems or relevant codes in the same Read chapter. Term descriptions include “H/O” (history of), and all dementia subtypes, were included.

## D

**Table D: Description of body system categories**

| Body system | Conditions |
| --- | --- |
| Inflammatory | Rheumatoid arthritis, psoriasis or eczema, inflammatory bowel disease, diverticulosis, prostate disorder |
| Cardiovascular | Stroke, hypertension, chronic heart disease, heart failure, atrial fibrillation, peripheral vascular disease |
| Neurological | Epilepsy, schizophrenia or bipolar disorder, migraine, Parkinson’s disease, learning disabilities, multiple sclerosis |
| Mental health | Anxiety or depression, psychoactive substance misuse, alcohol misuse, anorexia or bulimia |
| Respiratory | Asthma, chronic obstructive pulmonary disease, sinusitis, bronchiectasis |
| Gastrointestinal | Peptic ulcer, constipation, irritable bowel syndrome |
| Endocrine | Diabetes, thyroid dysfunction |
| Pain | Pain |
| Cancer | Cancer |
| Kidney | Chronic kidney disease |
| Liver | Chronic liver disease or hepatitis |
| Sensory | Hearing loss, blindness/low vision |

## E

**Table E: Risk of dementia in people with multimorbidity including each body system**

| **Body system** | **Cox regression**  **Unadjusted**  **HR [95% CI]** | **Cox regression Adjusted**  **HR [95% CI]** | **Competing risk of death Adjusted HR [95% CI]** |
| --- | --- | --- | --- |
| Inflammatory | 1.03 [1.01,1.05] | 0.95 [0.94,0.96] | 1.14 [1.12,1.16] |

| Cardiovascular | 0.96 [0.94,0.97] | 1.08 [1.03,1.06] | 1.22 [1.19,1.24] |
| --- | --- | --- | --- |

| Neurological | 2.59 [2.54,2.64] | 2.96 [2.90,3.02] | 2.19 [2.15,2.23] |
| --- | --- | --- | --- |

| Mental health | 1.87 [1.83,1.91] | 1.90 [1.87,1.94] | 1.40 [1.37,1.42] |
| --- | --- | --- | --- |

| Respiratory | 1.02 [0.99,1.03] | 0.95 [0.93,0.97] | 0.82 [0.80,0.83] |
| --- | --- | --- | --- |

| Gastrointestinal | 1.14 [1.12,1.15] | 1.18 [1.17,1.20] | 1.48 [1.46,1.50] |
| --- | --- | --- | --- |

| Endocrine | 1.17 [1.15,1.19] | 1.04 [1.03,1.06] | 1.14 [1.11,1.16] |
| --- | --- | --- | --- |

| Pain | 1.23 [1.15,1.32] | 0.88 [0.83,0.94] | 1.15 [1.08,1.23] |
| --- | --- | --- | --- |

| Cancer | 0.78 [0.76,0.79] | 0.77 [0.76,0.79] | 0.66 [0.65,0.68] |
| --- | --- | --- | --- |

| Kidney | 0.99 [0.98,1.01] | 0.47 [0.46,0.48] | 1.98 [1.95,2.01] |
| --- | --- | --- | --- |

| Liver | 1.44 [1.31,1.57] | 1.20 [1.10,1.31] | 0.73 [0.67,0.80] |
| --- | --- | --- | --- |

| Sensory | 0.88 [0.87,0.90] | 0.90 [0.89,0.92] | 1.34 [1.32,1.36] |
| --- | --- | --- | --- |

Exponentiated coefficients; 95% confidence intervals in brackets
Both models adjusted for sex, year of birth, deprivation, and residual multimorbidity
In comparison to multimorbidity in another body system
*Appendix D for list of conditions within each body system

## F

**Table F: Risk of dementia in people with multimorbidity including each individual condition**

| **Condition** | **Cox regression**  **Unadjusted**  **HR [95% CI]** | **Cox regression**  **Adjusted**  **HR [95% CI]** |
| --- | --- | --- |
| Alcohol misuse | 3.56 [3.37,3.76] | 2.80 [2.65,2.96] |

| Anorexia or bulimia | 1.62 [1.56,1.69] | 1.64 [1.57,1.71] |
| --- | --- | --- |

| Anxiety or depression | 2.32 [2.27,2.37] | 1.92 [1.88,1.96] |
| --- | --- | --- |

| Asthma | 1.22 [1.19,1.25] | 0.99 [0.97,1.01] |
| --- | --- | --- |

| Atrial fibrillation | 1.09 [1.08,1.12] | 0.89 [0.88,0.91] |
| --- | --- | --- |

| Blindness or low vision | 1.02 [0.99,1.05] | 1.07 [1.05,1.09] |
| --- | --- | --- |

| Bronchiectasis | 1.25 [1.17,1.33] | 0.87 [0.81,0.93] |
| --- | --- | --- |

| Cancer | 0.95 [0.93,0.97] | 0.84 [0.82,0.86] |
| --- | --- | --- |

| Coronary heart disease | 1.14 [1.12,1.16] | 1.06 [1.04,1.07] |
| --- | --- | --- |

| Chronic kidney disease | 1.23 [1.21,1.24] | 0.55 [0.54,0.55] |
| --- | --- | --- |

| Chronic liver disease or viral hepatitis | 1.70 [1.56,1.86] | 1.27 [1.16,1.38] |
| --- | --- | --- |

| Chronic sinusitis | 1.39 [1.33,1.45] | 0.97 [0.93,1.02] |
| --- | --- | --- |

| Constipation | 1.42 [1.40,1.44] | 1.30 [1.29,1.33] |
| --- | --- | --- |

| Chronic obstructive pulmonary disease | 1.13 [1.10,1.16] | 0.98 [0.96,1.01] |
| --- | --- | --- |

| Diabetes | 1.47 [1.43,1.49] | 1.15 [1.12,1.17] |
| --- | --- | --- |

| Diverticular disease of intestine | 1.23 [1.20,1.25] | 0.93 [0.91,0.95] |
| --- | --- | --- |

| Epilepsy | 2.60 [2.52,2.69] | 2.38 [2.29,2.46] |
| --- | --- | --- |

| Hearing loss | 1.17 [1.15,1.19] | 0.95 [0.94,0.97] |
| --- | --- | --- |

| Heart failure | 0.83 [0.81,0.85] | 0.89 [0.87,0.92] |
| --- | --- | --- |

| Hypertension | 1.35 [1.33,1.36] | 0.95 [0.94,0.97] |
| --- | --- | --- |

| Inflammatory bowel disease | 1.41 [1.32,1.50] | 1.13 [1.06,1.21] |
| --- | --- | --- |

| Irritable bowel syndrome | 1.48 [1.44,1.53] | 1.04 [1.01,1.07] |
| --- | --- | --- |

| Learning disability | 9.97 [9.16,10.8] | 7.50 [6.89,8.16] |
| --- | --- | --- |

| Migraine | 1.48 [1.31,1.67] | 0.99 [0.88,1.12] |
| --- | --- | --- |

| Multiple sclerosis | 2.23 [1.95,2.54] | 1.76 [1.54,2.00] |
| --- | --- | --- |

| Chronic pain | 1.47 [1.38,1.57] | 0.90 [0.84,0.96] |
| --- | --- | --- |

| Parkinson's disease | 2.82 [2.74,2.91] | 3.12 [3.03,3.22] |
| --- | --- | --- |

| Peptic ulcer disease | 1.28 [1.25,1.32] | 1.18 [1.15,1.21] |
| --- | --- | --- |

| Peripheral vascular disease | 1.13 [1.09,1.17] | 1.02 [0.99,1.06] |
| --- | --- | --- |

| Prostate disorders | 1.31 [1.28,1.34] | 1.10 [1.07,1.13] |
| --- | --- | --- |

| Psoriasis or eczema | 1.37 [1.32,1.43] | 1.11 [1.07,1.15] |
| --- | --- | --- |

| Psychoactive substance misuse | 2.19 [1.97,2.44] | 1.75 [1.57,1.95] |
| --- | --- | --- |

| Rheumatoid arthritis | 1.08 [1.05,1.11] | 0.90 [0.87,0.92] |
| --- | --- | --- |

| Schizophrenia or bipolar | 3.83 [3.71,3.955 | 4.52 [4.39,4.67] |
| --- | --- | --- |

| Stroke or TIA | 1.69 [1.66,1.71] | 1.82 [1.80,1.85] |
| --- | --- | --- |

| Thyroid disorders | 1.32 [1.30,1.35] | 1.05 [1.03,1.07] |
| --- | --- | --- |

Exponentiated coefficients; 95% confidence intervals in brackets

Adjusted Cox model adjusted for sex, year of birth, deprivation and residual multimorbidity.

In comparison to people with any other condition.

In people with multimorbidity, learning disability presented the greatest risk of dementia (HR=7.50, 95 % CI 6.89-8.16) in comparison to multimorbidity containing any other condition, followed by schizophrenia or bipolar disorder (HR=4.53, 95% CI 4.39-4.67) and Parkinson’s disease (HR=3.13, 95% CI 3.03-3.22). The pattern was retained but attenuated after accounting for the competing risk of death (Figure 2).

**
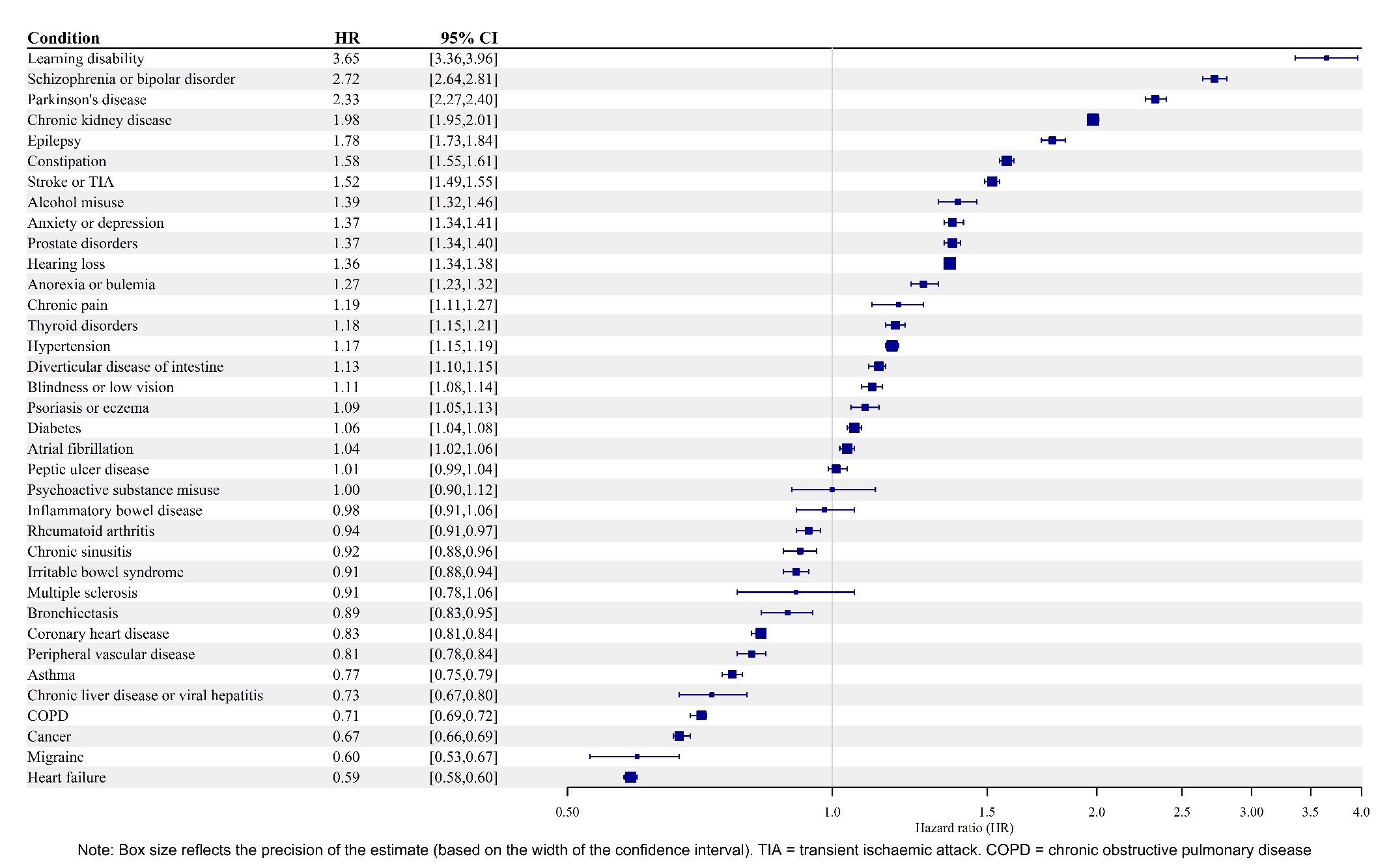

Figure F.1: Cox regression model reporting hazard ratio and 95% confidence interval for risk of multimorbidity by individual condition, adjusted for sex, year of birth, deprivation and residual multimorbidity. This is a competing risk of death model reporting the probability of a dementia diagnosis accounting for the competing risk of death by condition, in comparison to people with any other condition. Box size reflects the precision of the estimate (based on the width of the confidence interval). TIA = transient ischaemic attack; COPD = chronic obstructive pulmonary disease**

## G

**Table G: Description of people with multimorbidity by body system at follow-up start**

| Demographic | Inflammatory | Cardiovascular | Neurological | Mental health | Respiratory | Gastrointestinal |
| --- | --- | --- | --- | --- | --- | --- |
| N | 89,527 | 210,381 | 20,355 | 44,825 | 77,644 | 174,026 |
| Sex |  |  |  |  |  |  |
| Male | 54,396 (60.8%) | 106,895 (50.8%) | 8,994 (44.2%) | 17,247 (38.5%) | 38,184 (49.2%) | 82,576 (47.5%) |
| Female | 35,131 (39.2%) | 103,486 (49.2%) | 11,361 (55.8%) | 27,578 (61.5%) | 39,460 (50.8%) | 91,450 (52.5%) |
| Age at baseline in years (mean, SD) | 54.224 (18.8) | 55.492 (19.1) | 52.663 (19.5) | 47.761 (18.2) | 51.816 (18.7) | 54.247 (19.5) |
| Years of follow-up (mean, SD) | 23.067 (16.7) | 21.771 (16.9) | 19.584 (15.9) | 21.850 (15.5) | 21.560 (16.3) | 21.092 (16.5) |
| Region of England |  |  |  |  |  |  |
| North East | 2,283 (2.6%) | 5,215 (2.5%) | 509 (2.5%) | 1,513 (3.4%) | 2,142 (2.8%) | 4,510 (2.6%) |
| North West | 14,050 (15.7%) | 33,983 (16.2%) | 3,272 (16.1%) | 8,646 (19.3%) | 14,187 (18.3%) | 30,262 (17.4%) |
| Yorkshire & The Humber | 3,777 (4.2%) | 9,696 (4.6%) | 1,017 (5.0%) | 1,994 (4.4%) | 3,458 (4.5%) | 8,313 (4.8%) |
| East Midlands | 3,368 (3.8%) | 8,511 (4.0%) | 834 (4.1%) | 1,956 (4.4%) | 3,065 (3.9%) | 7,347 (4.2%) |
| West Midlands | 9,739 (10.9%) | 22,084 (10.5%) | 2,050 (10.1%) | 4,935 (11.0%) | 8,020 (10.3%) | 18,824 (10.8%) |
| East of England | 10,258 (11.5%) | 22,345 (10.6%) | 2,131 (10.5%) | 4,831 (10.8%) | 7,922 (10.2%) | 18,902 (10.9%) |
| London | 10,961 (12.2%) | 27,583 (13.1%) | 2,821 (13.9%) | 5,164 (11.5%) | 10,132 (13.0%) | 21,251 (12.2%) |
| South East | 25,008 (27.9%) | 57,022 (27.1%) | 5,364 (26.4%) | 10,743 (24.0%) | 20,413 (26.3%) | 45,443 (26.1%) |
| South West | 10,083 (11.3%) | 23,942 (11.4%) | 2,357 (11.6%) | 5,043 (11.3%) | 8,305 (10.7%) | 19,174 (11.0%) |
| Patient-level deprivation |  |  |  |  |  |  |
| Deciles 1+2: least deprivation | 21,816 (24.4%) | 46,175 (21.9%) | 4,273 (21.0%) | 8,820 (19.7%) | 15,989 (20.6%) | 37,742 (21.7%) |
| Deciles 3-8: middle 60% | 54,460 (60.8%) | 128,407 (61.0%) | 12,276 (60.3%) | 26,707 (59.6%) | 46,515 (59.9%) | 105,255 (60.5%) |
| Deciles 9+10: most deprivation | 13,251 (14.8%) | 35,799 (17.0%) | 3,806 (18.7%) | 9,298 (20.7%) | 15,140 (19.5%) | 31,029 (17.8%) |

Table G continued

| Demographic | Endocrine | Pain | Cancer | Kidney | Liver | Sensory |
| --- | --- | --- | --- | --- | --- | --- |
| N | 79,416 | 3,972 | 340,282 | 125,512 | 3,777 | 80,681 |
| Sex |  |  |  |  |  |  |
| Male | 34,289 (43.2%) | 1,573 (39.6%) | 162,632 (47.8%) | 58,670 (46.7%) | 2,025 (53.6%) | 41,981 (52.0%) |
| Female | 45,127 (56.8%) | 2,399 (60.4%) | 177,650 (52.2%) | 66,842 (53.3%) | 1,752 (46.4%) | 38,700 (48.0%) |
| Age at baseline in years (mean, SD) | 53.652 (19.1) | 50.164 (18.0) | 53.047 (19.3) | 52.782 (18.9) | 48.817 (17.1) | 56.056 (19.4) |
| Years of follow-up (mean, SD) | 21.402 (16.7) | 21.269 (15.6) | 20.790 (16.4) | 23.428 (16.6) | 19.349 (15.4) | 23.048 (16.7) |
| Region of England |  |  |  |  |  |  |
| North East | 1,809 (2.3%) | 103 (2.6%) | 8,155 (2.4%) | 2,177 (1.7%) | 104 (2.8%) | 2,124 (2.6%) |
| North West | 12,789 (16.1%) | 748 (18.8%) | 53,302 (15.7%) | 17,182 (13.7%) | 666 (17.6%) | 13,253 (16.4%) |
| Yorkshire & The Humber | 3,236 (4.1%) | 129 (3.2%) | 15,532 (4.6%) | 2,777 (2.2%) | 162 (4.3%) | 3,581 (4.4%) |
| East Midlands | 3,023 (3.8%) | 117 (2.9%) | 13,762 (4.0%) | 1,885 (1.5%) | 153 (4.1%) | 3,050 (3.8%) |
| West Midlands | 8,334 (10.5%) | 394 (9.9%) | 35,299 (10.4%) | 14,620 (11.6%) | 303 (8.0%) | 8,805 (10.9%) |
| East of England | 8,253 (10.4%) | 277 (7.0%) | 36,595 (10.8%) | 10,890 (8.7%) | 331 (8.8%) | 8,634 (10.7%) |
| London | 11,242 (14.2%) | 595 (15.0%) | 45,720 (13.4%) | 21,562 (17.2%) | 650 (17.2%) | 9,644 (12.0%) |
| South East | 21,831 (27.5%) | 1,211 (30.5%) | 93,848 (27.6%) | 43,321 (34.5%) | 1,015 (26.9%) | 22,025 (27.3%) |
| South West | 8,899 (11.2%) | 398 (10.0%) | 38,069 (11.2%) | 11,098 (8.8%) | 393 (10.4%) | 9,565 (11.9%) |
| Patient-level deprivation |  |  |  |  |  |  |
| Deciles 1+2: least deprivation | 16,701 (21.0%) | 710 (17.9%) | 77,524 (22.8%) | 33,998 (27.1%) | 787 (20.8%) | 19,167 (23.8%) |
| Deciles 3-8: middle 60% | 48,885 (61.6%) | 2,462 (62.0%) | 206,091 (60.6%) | 74,723 (59.5%) | 2,243 (59.4%) | 48,708 (60.4%) |
| Deciles 9+10: most deprivation | 13,830 (17.4%) | 800 (20.1%) | 56,667 (16.7%) | 16,791 (13.4%) | 747 (19.8%) | 12,806 (15.9%) |
| Note: this table contains only people with multimorbidity. People can be present in more than one body system. | | | | | | |

## H

**Table H.1: Risk of dementia by total number of conditions before dementia**

| **Number of conditions** | | **Cox regression Adjusted**  **HR [95% CI]** | **Competing risk of death Adjusted**  **HR [95% CI]** |
| --- | --- | --- | --- |
| 2 conditions | 1.66 [1.61,1.69] | | 2.64 [2.58,2.70] |
| 3 conditions | 2.14 [2.09,2.19] | | 3.61 [3.53,3.69] |
| 4 conditions | 2.07 [2.02,2.11] | | 4.31 [4.21,4.40] |
| 5+ conditions | 1.92 [1.88,1.96] | | 5.27 [5.17,5.36] |

Exponentiated coefficients; 95% confidence intervals in brackets

Both models adjusted for sex, year of birth, and deprivation
In comparison to no multimorbidity (0-1 conditions)

Any conditions within 12 months before dementia diagnosis excluded to mitigate risk of reverse causality

**Table H.2: Risk of dementia by total number of conditions already present at baseline**

| **Number of conditions** | **Cox regression**  **Unadjusted**  **HR [95% CI]** | **Cox regression**  **Adjusted**  **HR [95% CI]** |
| --- | --- | --- |
| 2 conditions | 3.53 [3.38,3.69] | 2.89 [2.77,3.03] |
|  |  |  |
| 3 conditions | 8.14 [7.77,8.52] | 6.59 [6.29,6.90] |
|  |  |  |
| 4 conditions | 13.12 [12.44,13.83] | 10.26 [9.73,10.83] |
|  |  |  |
| 5+ conditions | 21.72 [20.67,22.83] | 16.12 [15.32,16.95] |

Exponentiated coefficients; 95% confidence intervals in brackets

Both models adjusted for sex, year of birth and deprivation.

In comparison to people without multimorbidity (0-1 conditions)

Dementia diagnoses already present and within 12 months of baseline excluded

It was not possible to run competing risk of death analyses for number of conditions already present at baseline, as any patients who died before baseline were excluded from the source population under the criteria as having no available follow-up time.
